# Distal muscle weakness as the main onset symptom in thymoma-associated myasthenia gravis: a case report and literature review

**DOI:** 10.3389/fimmu.2025.1498847

**Published:** 2025-01-24

**Authors:** Xuan Wu, Xiao-tian Xu, Lin Zhou, Kai Qiao, Chong-bo Zhao, Su-shan Luo

**Affiliations:** ^1^ Department of Neurology, Affiliated Hospital of Yangzhou University, Yangzhou, China; ^2^ Department of Neurology, Huashan Hospital, Fudan University, Shanghai, China

**Keywords:** myasthenia gravis, autoimmune disorder, neuromuscular disease, distal muscle weakness, thymoma

## Abstract

Myasthenia gravis (MG) is an autoimmune disorder within the spectrum of neuromuscular rare diseases, characterized by fluctuating muscle weakness. This report presents a case of a middle-aged woman with a chronic onset of asymmetric upper limb weakness accompanied by difficulty in finger extension, without ptosis or fluctuation for 4 years. The patient was finally diagnosed with MG by a significant decrement of Compound Muscle Action Potential in repetitive nerve stimuli, positive anti-acetylcholine receptor antibodies as well as the presence of a mass located in the anterior mediastinum. With subsequent immunotherapies for one month, the patient exhibited marked enhancement in muscle strength, followed by an uneventful thymectomy. After two months, the patient’s symptoms were fully alleviated, as evidenced by the reduction in Quantitative MG Score from 9 to 4 points, Myasthenia Gravis Composite Score from 6 to 1 points, Myasthenia Gravis Activities of Daily Living Score from 4 to 1 points, and Myasthenia Gravis Quality of Life-15 score from 14 to 8 points respectively. This case highlights the importance of differentiating autoimmune disorders from hereditary neuromuscular diseases and initiating timely treatment.

## Introduction

Myasthenia gravis (MG) is a rare autoimmune neuromuscular disorder characterized by fluctuating muscle weakness. This weakness can affect the extraocular muscles, limbs, bulbar region, and respiratory muscles (1). Approximately 15 to 50 percent of patients with MG showed ocular involvement at onset, such as ptosis and diplopia; however, atypical muscle presentations are possible and can make a prompt diagnosis difficult (2). In fact, distal weakness of the upper limbs is occasionally noted in patients with MG and could be misdiagnosed with myopathy or other neurological conditions (3). Although the pathogenesis of MG involves the production of autoantibodies against the acetylcholine receptors (AChR) at the neuromuscular junction, leading to impaired neuromuscular transmission and muscle weakness, the mechanisms underlying MG’s asymmetric or focal forms remain less understood. It is important to recognize these atypical presentations, as they may lead to diagnostic delays and improper management if not promptly identified (4).

In this study, we present a case with generalized MG presenting with asymmetric upper limb weakness and difficulty in extending the fingers as the main symptoms. Through a comprehensive clinical evaluation and diagnostic workup, we aim to characterize this atypical presentation of MG further and discuss the implications for diagnosis and management.

## Case report

The patient is a 45-year-old Han Chinese female who presented to the Department of Neurology at Huashan Hospital, affiliated with Fudan University, with a chief complaint of “left distal upper limb weakness for 4 years, and limb muscle weakness for 6 months.” The patient has experienced progressive weakness in the left shoulder since 2019, with no significant fluctuation in the symptoms. She noticed muscle atrophy in her left upper arm but did not pay much attention to it as it did not affect her daily activities. In July 2022, she began to experience difficulty combing her hair and raising her left arm, which worsened after activity and improved with rest. A month later, she noticed drooping of the middle and the ring fingers on her right hand, significantly affecting the fine motor movements of the fingers. In August, she started to feel heaviness in bilateral lower limbs, difficulty in climbing stairs and squatting, and shortened breath after activity. These symptoms recurred intermittently without receiving formal treatment. She reported progressive worsening in lifting the head, breathing and raising the limbs after COVID-19 infection in December 2022.

Throughout the illness, she did not lose weight, nor experience dysphagia or other bulbar symptoms, with no sensory abnormalities, muscle spasms, contractures, muscle pain, changes in urine color, rashes, difficulty swallowing, speech difficulties, or recent exposure to new medications or toxins. There was no significant family history or personal history. Skin color and temperature appear normal, with no abnormalities in skeletal system development. Vital signs are stable, and no other systemic signs or symptoms were detected. The patient is conscious with no ptosis, normal eye movements, and a light reflex sensitivity. Speech is clear, and there are no signs of scoliosis, winged scapula, high arches, joint contractures, or muscle rigidity. According to the Medical Research Council (MRC) muscle strength assessment, there is a noticeable decrease in muscle strength in lifting the left upper limb (4-/3) and extending fingers on the right side (3/5-). There is severe weakness in the proximal lower limbs (MRC 3/4), and the distal lower limbs are at a level of 5. Furthermore, the weakness in the proximal limbs was not fixed and worsened with exercise. Overall, the patient exhibits normal muscle tone, symmetric reflexes in all limbs (+), accurate finger-nose and heel-knee-shin tests, normal sensory perception to pinprick and temperature in all limbs, normal vibratory sensation with a tuning fork, and an absence of pathological signs and meningeal irritation signs.

### Diagnostic evaluation

The patient presented with a slow progression of symptoms, initial asymmetrical distal weakness in the upper limbs, and proximal fluctuating muscle weakness in the four limbs. Deep tendon reflexes were symmetrically brisk (+), with no detectable pathological signs. The lesion can be localized to the lower motor neuron pathway, involving the anterior horn of the spinal cord, nerve roots, peripheral nerves, neuromuscular junctions, or muscles. Due to the absence of sensory abnormalities, nerve root pain, or fasciculations, the differential diagnosis primarily focuses on muscle pathology, with atypical neuromuscular junction disorders not being ruled out.

### Qualitative diagnosis

The patient is a middle-aged female with a chronic muscle weakness for 4 years, starting from the distal limbs. Although no family history was reported, genetic and acquired etiologies are both possible. Based on the localization, if the pathology is myogenic, the differential diagnosis includes distal myopathies, neutral lipid storage diseases, or inflammatory myopathies. If the condition involves neuromuscular junctions, atypical myasthenia gravis (MG) or Lambert-Eaton syndrome should be considered.

Complete Blood Count, Liver and Kidney Function, Thyroid Function, Cardiac Biomarkers, serum Light Chains, serum and urine immunofixation electrophoresis, Tumor biomarkers, Folate, Complete Vitamin Panel, Blood Sugar, and Glycated Hemoglobin were all within normal limits. Serum Creatine Kinase was 175 U/L (Range 50-310 U/L), and Lactate Dehydrogenase was 197 U/L (Range 120-250 U/L). Human Immunodeficiency Virus Antibodies and Syphilis Specific Antibodies were negative. Lumbar Puncture cerebrospinal fluid analysis showed white blood cells at 3.2×10^6/L (Range 0-8×10^6/L) and protein at 376 mg/L (Range 150-450 mg/L). Autoimmune Antibody Panel (including anti-nuclear antibody spectrum, anti-mitochondrial antibodies, anti-phospholipid antibodies, ENA antibody spectrum, anti-neutrophil cytoplasmic antibodies, etc.), Myositis-related Antibody Panel (including SRP, HMGCR, Jo-1, Ro-52, NXP2, MDA5, Mi-2, etc.), and Anti-Voltage-Gated Calcium Ion Channel (VGCC) Antibodies were all within normal limits. Anti-AChR IgG was 166.8 nmol/L, exceeding the normal range of <0.4 nmol/L by ELISA.

The nerve conduction testing revealed normal motor and sensory nerve conduction velocities and amplitudes, with no significant conduction blocks or waveform dispersion observed. Needle electromyography indicated increased spontaneous potentials such as fibrillation potentials and positive sharp waves in the partially examined muscles of the left upper limb. During light contraction, motor unit potentials (MUPs) appeared narrow or partially narrowed, with early recruitment observed during strong contraction, indicating neuromuscular junction (NMJ) blockade. Low-frequency (3Hz) repetitive nerve stimulation of the upper limb and facial muscles resulted in a greater than 15% decrease in compound muscle action potential (CMAP) amplitudes. Following a 10-second strong muscle contraction, the increase in CMAP amplitude did not exceed the normal range (**Table 1**). Based on the comprehensive results of the electrophysiological examinations, a dysfunction at the neuromuscular junction is considered.

**Table 1:** Results of repetitive nerve stimulation.

Repetitive electrical stimulation	Frequency	Amplitude decrement %	Area decrement%
Abductor of left little finger	10@3HZ	-5.9	-6.9
left deltoid	10@3HZ	-35.5	-39.1
Orbicularis muscle of the left eye	10@3HZ	-34.9	-72.6
Right trapezius muscle	10@3HZ	-36.0	-44.0
Right extensor digitorum longus	10@3HZ	-6.2	-7.7

### Radiological studies

**Figure 1:**
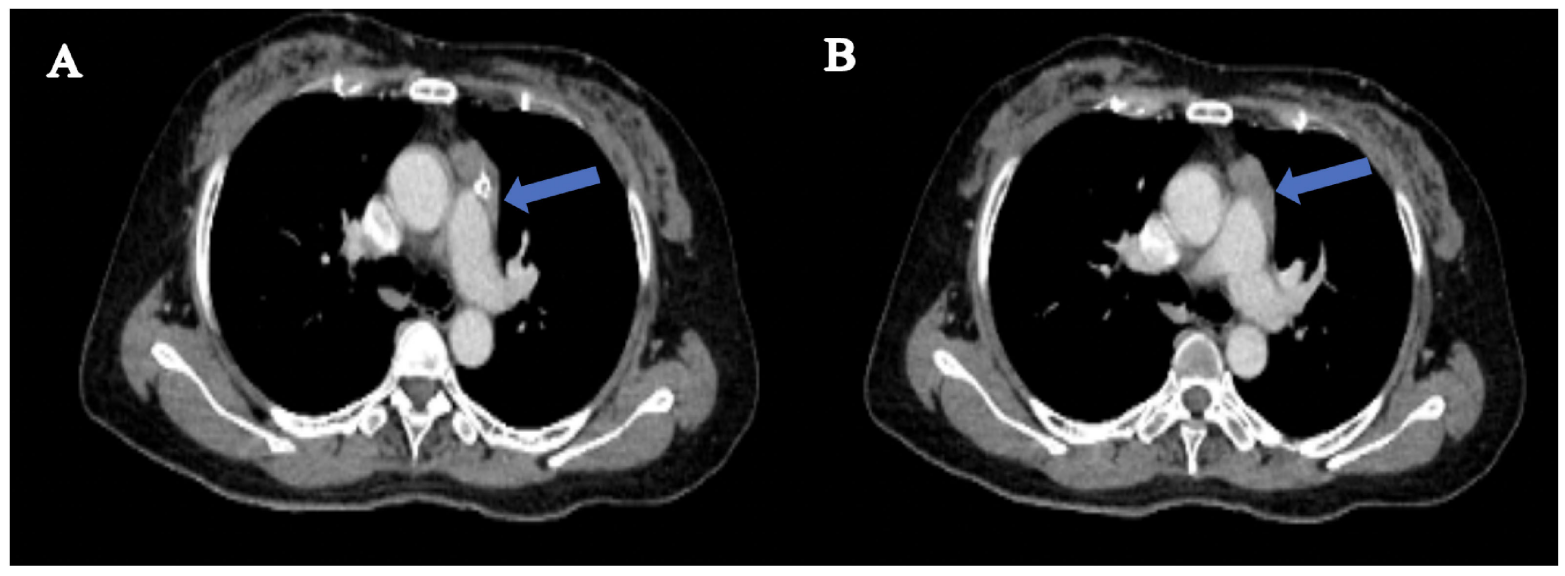
Radiological findings in thymus-enhanced CT scan. **(A)** a mass-like soft tissue density shadow is observed in the left upper anterior mediastinum. **(B)** shows homogeneous enhancement of the soft tissue in the upper anterior mediastinum, with smooth borders and no invasion of surrounding major blood vessels or the heart.

A conventional MRI scan of the head and cervical spine revealed no apparent abnormalities. An enhanced CT scan of the thymus showed a mass-like soft tissue density in the left upper anterior mediastinum, measuring approximately 37mm×18mm, with clear borders, suggesting a possible thymoma (**Figure 1**). A muscle MRI with both conventional scans and STIR (Short Tau Inversion Recovery) sequences showed no evidence of muscle atrophy, fatty infiltration, or changes in muscle signal.

### Diagnosis and treatment

**Figure 2:**
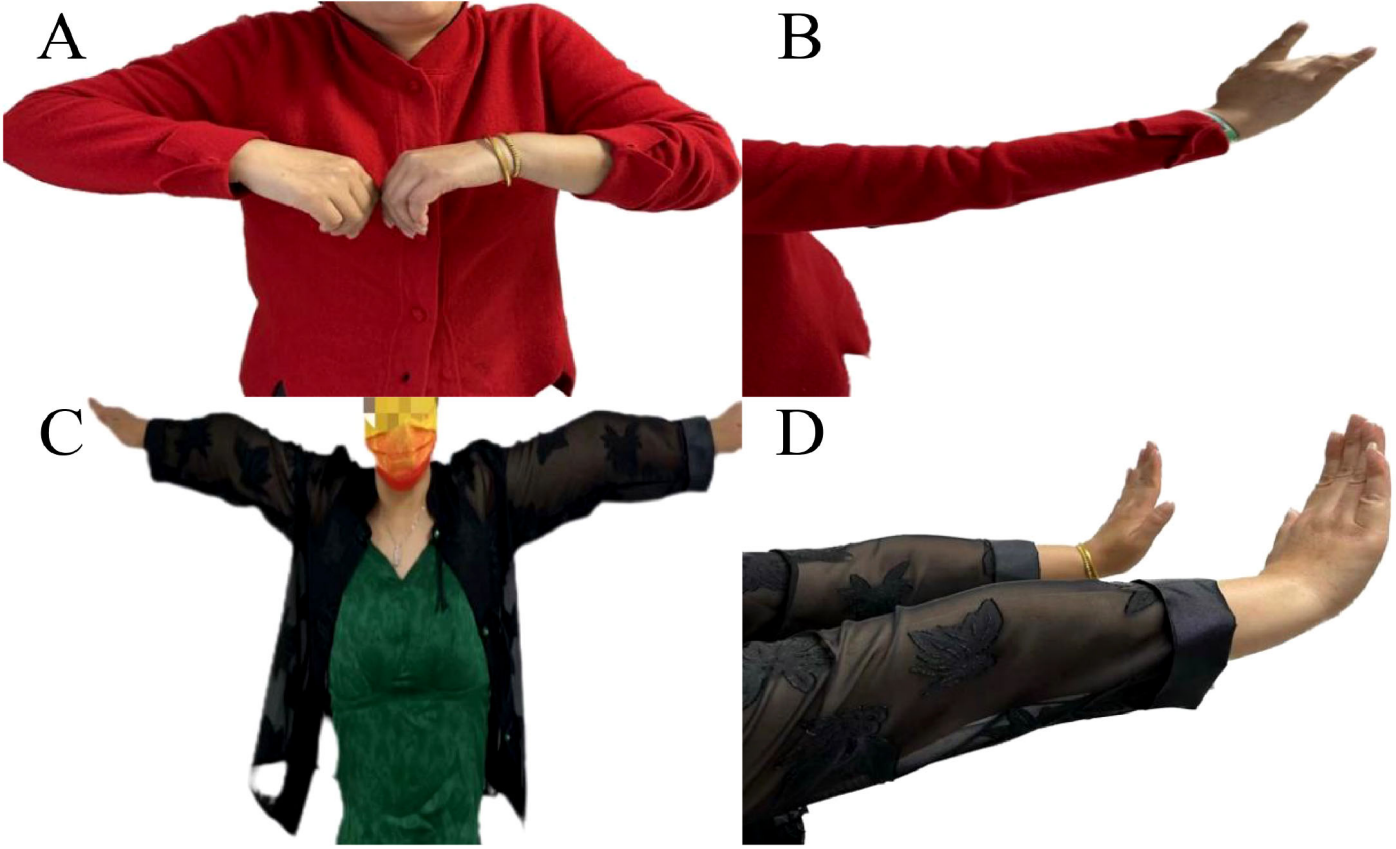
Illustration of improvement in upper limb weakness following immunotherapy and thymoma resection. Upon admission, the patient presented weakness in raising the left upper limb **(A)** and difficulty extending the fingers of the right hand, particularly the third and fourth fingers **(B)**. Following adequate immunotherapy and thymoma resection, muscle strength in the left upper limb **(C)** and finger extension in the right hand **(D)** significantly improved.

According to the diagnosis criteria of International Consensus Guidance for Management (5), the final diagnosis was MG with Myasthenia Gravis Foundation of America (MGFA) Type IIa (**Figure 2A**). Before starting treatment, the patient’s MG clinical scores were as follows: QMG 9 points, MGC 6 points, MG-ADL 4 points, and MG-QOL15 14 points.

The patient was prescribed oral prednisolone acetate tablets at a dose of 20 mg once daily, along with pyridostigmine 60 mg three times a day. Following two months of treatment, muscle strength was significantly improved (MRC 4+/5), and the scores on the MG-related scales QMG, MGC, MG-ADL, and MG-QOL15 were 4, 1, 1, and 8 respectively. In May 2023, the patient underwent thymoma resection, with pathology results indicating Type B2. Postoperatively, the patient continued to take oral prednisolone at a dose of 20 mg once daily (**Figures 2B, C**). Then, the prednisolone regimen entails a gradual reduction of 5 mg every four weeks, culminating in a maintenance dosage of 5 mg per day. Subsequently, an alternate-day dosing schedule, consisting of 5 mg administered every second day, is implemented, and pyridostigmine 60 mg three times a day. At the 2-month follow-up, the patient’s symptoms had completely resolved (MRC 5) (**Table 2**, **Figure 2D**).

**Table 2:** Changes in MG-associated clinical scores before and after treatment.

Clinical Variables	Before treatment	After treatment
QMG	9	4
MGC	6	1
MG-ADL	4	1
MG-QOL15	14	8
MRC(left/right)		
Neck flexion	3	5-
shoulder abduction	3/4-	4/5-
elbow flexion and extension	4-/4	4/5-
wrist flexion and extension	4/4-	5/5
finger flexion	4/4	5/5
finger extension	4/3	5/5-
hip flexion	3/4	4/5
hip extension	4-/4	5/5
knee flexion and extension	4-/4	5/5
foot dorsiflexion and plantarflexion	5/5	5/5

QMG, Quantitative MG Score; MGC, MG Composite score; ADL, Activities of Daily Living score; QOL, Quality of Llife Score; MRC, Medical Research Council Score.

## Literature review

We searched the terms “distal limb muscle” and “myasthenia gravis” and identified 41 patients from 32 case reports through Embase, MEDLINE, Scopus and CNKI databases from January 1990 to June 2024 (**Supplementary Table S1**). Apart from 2 patients with no documented thymus status, we finally included 39 patients for further analysis. We stratified them into two groups: thymoma-associated MG (TAMG) (n=28) and non-thymoma-associated MG (non-TAMG) (n=11). The diagnostic age and sex were comparable in these two groups. Preferential involvement in bulbar muscle was significant in non-TAMG, as compared to TAMG (63.6% versus 14.3%, *P*=0.004). No significant differences were identified in other muscles, including ocular, bulbar, neck and respiratory muscles. The thymoma group showed a broader range of antibody combinations. (**Supplementary Table S2**).

## Discussion

MG is a rare but treatable autoimmune disease. A review of 63 studies spanning from the mid-1950s to 2020, encompassing a participant pool of over 1.2 billion individuals with a global prevalence rate of 124 cases per million people (6). MG is linked to a substantial health burden, manifesting as heightened medical costs, reduced productivity, and a diminished quality of life, primarily due to the debilitating muscle weakness and the adverse effects of treatment medications (7). Therefore, there is an urgent need for attention to this disease for prevention and treatment. MG is an autoimmune condition that impairs NMJ transmission, leading to varying muscle weakness. Typical diagnosis involves electrophysiological and antibody tests. A rare presentation with asymmetric distal weakness in the hands can complicate diagnosis, necessitating awareness by medical professionals for timely and effective treatment. This case report underscores MG’s atypical symptoms and aids in distinguishing it from other neuromuscular disorders.

The co-occurrence of MG and muscle atrophy has been documented in the literature (8). This concurrent presentation is uncommon and is hypothesized to stem from a combination of factors, including the chronicity of MG itself, the myopathic effects of sustained glucocorticoid therapy at moderate to high doses, and the presence of antibodies targeting the muscle-specific receptor tyrosine kinase in MG patients (9). The diagnosis of classic MG with anti-AChR antibodies is typically straightforward. However, some cases of MG have been reported with atypical and unusual patterns of muscle weakness, particularly involving distal and asymmetric muscle weakness. In cases of moderate to severe or untreated disease, muscle weakness may become fixed without fluctuations, leading to diagnostic confusion (3). Considering the asymmetric/distal weakness of the upper limbs with normal sensory systems and tendon reflexes, a lower motor neuron lesion should be considered.

Atypical distal muscle weakness is not common in MG, with less than 10% of MG patients experiencing weakness in the hands or forearm muscles, making it relatively rare (10). Among the asymmetrical distribution of limb weakness, the most commonly affected muscles are the finger extensors (10, 11), although the underlying reasons for this potential selectivity remain unclear. Nations et al. (12) found that out of 236 patients with MG diagnosis, 9 patients (3%) exhibited distal MG manifestations, particularly affecting the finger extensors compared to distal leg and foot muscles. Werner et al. (13) identified 6 out of 84 MG patients (7%) presenting primarily with distal limb muscle weakness and fatigue, with 5 of them mainly affected in the extensors of the hand and forearm. In a single-center study in China (14) that included 52 confirmed MG patients, 4 cases (7.7%) were classified as distal type. These patients developed distal limb muscle weakness asymmetrically within 1 month to 6 years after the onset of initial symptoms.

Compared to proximal muscles in terms of electrophysiology, repetitive nerve stimulation in distal muscles showed a more significant decrement, with a slight decrease in baseline Compound Muscle Action Potential (CMAP) amplitude. The present case exhibited typical features of “myopathy” on routine needle electromyography, highlighting the necessity for repeat nerve stimulation studies in patients undergoing electromyography for suspected myopathy (15). There is a significant correlation between the electrophysiological characteristics of Myasthenia Gravis (MG) and the severity of the disease (16). Specifically, the reduction in the amplitude of Compound Muscle Action Potential (CMAP) during Repetitive Nerve Stimulation (RNS) is strongly associated with the severity of MG. Additionally, myopathic changes, including “pseudomyopathy,” are not uncommon in MG patients and are often attributed to severe neuromuscular transmission dysfunction, which typically indicates a more severe disease state.

Thymomas display a spectrum of clinical presentations, ranging from being incidentally identified without any symptoms during routine diagnostic assessments, to causing localized symptoms or even mimicking the signs of MG and other neoplastic conditions (17). It’s important to note that 10% to 15% of MG patients also have a thymoma, and 30% to 45% of patients with thymoma develop MG. While thymoma recurrence can lead to MG worsening, it doesn’t impact the long-term prognosis. However, patients with inoperable tumors face a poorer prognosis for MG (18). This case is particularly remarkable for its lack of early symptoms, which is unusual given the tumor’s considerable size. The remarkable aspect of this case is that the MG symptoms were completely resolved after surgery, obviating the need for any subsequent medical therapy.

In addition to the common muscle weakness associated with MG, there are less common, yet significant, atypical presentations (19). These include conditions such as dropped foot, isolated biceps brachii weakness, dropped head syndrome with oculo-bulbar involvement, acute facial palsy, limb-girdle MG, MG with frequent falls, sphincter dysfunction, pseudo-paralytic MG, atrophic MG, and blepharospasm-like MG (20–23). Although these atypical forms constitute less than 5% of MG cases, they pose a substantial risk for misdiagnosis or delayed diagnosis. The time from symptom onset to diagnosis for the typical MG is generally 1-6 months, but for limb-girdle MG, it can extend to 1-15 years (19). Therefore, recognizing these rare phenotypes is essential for timely and accurate clinical diagnosis.

Currently, the treatment of MG is primarily based on traditional medications; however, there is an increasing number of targeted drugs designed for specific MG targets, such as rituximab, eculizumab, and batoclimab (24). Previous limited studies have suggested that atypical MG is similar to classical MG in terms of age, gender, disease presentation, effectiveness of treatment strategies, and prognosis (19). However, due to the limited number of cases in the atypical categories of MG, there are still some clinical issues that require further exploration. Additionally, more multicenter clinical studies will be necessary in the future to validate these findings.

The current study offers significant insights into the relationship between thymoma and MG, particularly focusing on the clinical features and therapeutic outcomes of patients suffering from limb muscle weakness. The observed changes in the antibody profile, particularly the emergence of shared or specific antibodies such as GAD, RYR, Titin, Agrin, and ANA, suggest a shift in the pathophysiology of MG over time. These molecular alterations could reflect changes in the signaling pathways involved in the autoimmune response targeting the NMJ. For instance, previous research highlighted the prevalence of acetylcholine receptor antibodies in MG patients, but our study extends this knowledge by revealing shifts in antibody status over time, suggesting potential alterations in the underlying disease mechanisms or treatment responses (25).

The implications of these findings for clinical practice are substantial. The observed increase in antibody-negative patients could signal a need for revised diagnostic criteria and treatment protocols, particularly in cases where traditional antibody testing is insufficient for accurate diagnosis. Such changes could lead to more personalized treatment approaches, enabling clinicians to tailor interventions based on individual immunological profiles rather than relying solely on conventional antibody tests. Furthermore, the study’s results underscore the importance of ongoing monitoring of antibody status as part of routine clinical evaluations, which could enhance patient outcomes and optimize therapeutic strategies. This potential shift in clinical practice could significantly impact the management of MG patients, particularly those with thymoma, thereby improving their overall prognosis and quality of life (26).

The final diagnosis of neuromuscular diseases is not determined from just an isolated anatomical site, but rather begins with the patient’s chief complaints and a physical examination. When analyzing a case of limb weakness at onset, the focus should initially be on the upper motor neuron pathway and/or lower motor neuron pathway before narrowing down to a more precise localization within the lower motor neuron pathway. Subsequently, a qualitative diagnosis of acquired or genetic etiology is made, distinguishing between treatable diseases and those requiring genetic counseling or follow-up. Neurologists specializing in the diagnosis and treatment of rare diseases need to strengthen their basic skills in clinical localization and qualitative diagnosis, which may enhance their clinical diagnostic and treatment capabilities.

## Data Availability

The datasets presented in this article are not readily available because of ethical and privacy restrictions. Requests to access the datasets should be directed to the corresponding author.
