# Supplementary material for: Distal muscle weakness as the main onset symptom in thymoma-associated myasthenia gravis: a case report and literature review
Source: Front Immunol. 2025 Jan 24;16:1498847. doi: 10.3389/fimmu.2025.1498847 (PMC11802489; doi:10.3389/fimmu.2025.1498847)
Supplement: Supplementary file 1 [file Table1.docx]

**Supplementary Table 1.** Clinical characteristics of 39 distal myasthenia gravis patients and limb weakness in case reports.

| References | Sex | Age | ocular muscles | bulbar muscles | neck muscles | respiratory muscles | limb muscles | Antibodies relative to MG | RNS/EMG | Prognosis | Thymoma |
| --- | --- | --- | --- | --- | --- | --- | --- | --- | --- | --- | --- |
| Miao et al. 1 (2023)^[1]^ | M | 47 | NO | NO | NO | Yes | Yes | AChR, RYR, Titin | NA | Death | Yes |
| Miao et al. 2 (2023) | F | 35 | Yes | Yes | NO | Yes | Yes | AChR, Titin | myogenic damage, normal RNS | Remission | Yes |
| Monstad et al. (2009)^[2]^ | F | 49 | Yes | NO | NO | NO | Yes | AChR | NA | Remission | Yes |
| Evoli et al. (1999)^[3]^ | M | 32 | Yes | NO | NO | NO | Yes | AChR | EMG showed doublet, triplet, or multiplet discharges. RNS of right aux. & median nerves showed CMAP decrement of 26% & 13%. | Remission | Yes |
| Zhang et al. (2023)^[4]^ | F | 68 | Yes | NO | NO | NO | Yes | ANA(+);AChR,MuSK,LRP4(-) | Changes mainly in the proximal muscles, and compression of the bilateral median nerve wrists. | Remission | Yes |
| Antonio et al. (2023)^[5]^ | M | 47 | NO | NO | NO | NO | Yes | AChR | decremental response in both proximal and distal muscles | Remission | Yes |
| Wang et al. (2021)^[6]^ | M | 44 | NO | NO | NO | NO | Yes | Agrin | NA | Remission | Yes |
| Chihiro et al. (2021)^[7]^ | F | 77 | Yes | NO | NO | NO | Yes | AChR, Titin | NA | Remission | Yes |
| Reddy et al. (2020)^[8]^ | F | 68 | NO | NO | NO | NO | Yes | AChR | facial nerves and trapezius showed 42% decrement | Partial Remission | Yes |
| Mitesh et al. (2020)^[9]^ | M | 30 | Yes | NO | Yes | NO | Yes | AChR, GAD | NA | Partial Remission | Yes |
| Hoang et al. (2020)^[10]^ | M | 38 | NO | NO | NO | NO | Yes | AChR | NA | Remission | Yes |
| Dahal et al. (2019)^[11]^ | F | 45 | NO | NO | NO | Yes | Yes | AChR | NA | Remission | Yes |
| Kazuhiro et al. (2006)^[12]^ | M | 33 | NO | NO | NO | NO | Yes | AChR | Fasciculations, doublets, multiplets, and neuromyotonic discharges in the legs | Remission | Yes |
| Yoshihiro et al. (1991)^[13]^ | M | 54 | NO | NO | NO | NO | Yes | AChR | Fasciculation potentials with polyphasic motor units at rest. Right deltoid showed waning. Surface EMG showed resting motor unit potentials. | Partial Remission | Yes |
| Liu et al. (2022)^[14]^ | F | 54 | NO | NO | NO | NO | Yes | AChR, Titin | spontaneous potentials in the biceps brachii, deltoid, and paraspinal muscles | Partial Remission | Yes |
| Jiang et al. 1(2024)^[15]^ | M | 49 | Yes | NO | Yes | NO | Yes | AChR | EMG showed abnormal spontaneous potentials in muscles, with SFEMG Jitter >40μs in extensor digitorum, and RNS decrement ≥15% in facial, accessory, and axillary nerves. | Remission | Yes |
| Jiang et al. 2(2024) | F | 62 | Yes | NO | Yes | NO | Yes | AChR, Titin | EMG, SFEMG, and RNS indicate myogenic damage in both the upper and lower limbs. | Remission | Yes |
| Jiang et al. 3(2024) | F | 41 | Yes | NO | NO | NO | Yes | AChR | EMG shows myogenic damage with myotonic potentials in extensor digitorum communis, tibialis anterior, and antagonist muscles. | Death | Yes |
| Tatsuya et al. (2018)^[16]^ | F | 47 | NO | NO | NO | NO | Yes | AChR | NA | Death | Yes |
| Our case | M | 45 | NO | NO | NO | NO | Yes | AChR | Spontaneous potentials rose in left upper limb muscles, with >15% CMAP amplitude decrease after RNS. | Remission | Yes |
| Uncini A et al.(1997)^[17]^ | F | 69 | NO | NO | NO | NO | Yes | AChR | ADM: 10%; EDB: 33% with 3 Hz | Remission | No |
| Janssen JC et al.(1998)^[18]^ | M | 76 | Yes | Yes | NO | NO | Yes | AChR negative | ADM: 40%; 20% immediately after exer  cise; 65% 2 minutes post-exercise  with 3 Hz | Remission | No |
| Nations et al.(1999)^[19]^ | F | 28 | Yes | Yes | Yes | NO | Yes | AChR | Right accessory nerve: 24% with 3 Hz;  29% post-exercise with 3 Hz | Remission | No |
| Nations et al.(1999) | M | 60 | Yes | Yes | Yes | NO | Yes | AChR | Left ulnar nerve: 47% with 3 Hz; 66%  post-exercise with 3 Hz | Partial Remission | No |
| Gilad R et al.(2000)^[20]^ | F | 43 | NO | NO | NO | NO | Yes | AChR | Occasional fibrillation potentials in the anterior tibial muscles (foot toe) | Remission | No |
| Musser WS et al.(2001)^[21]^ | F | 42 | Yes | Yes | NO | NO | Yes | AChR | Both ulnar and median nerve:>50% with 2 Hz | Partial Remission | Yes |
| Musser WS et al.(2001) | F | 37 | Yes | Yes | NO | NO | Yes | AChR | Both ulnar and median nerve:>50% with 2 Hz | Partial Remission | Yes |
| Karacostas D et al.(2002)^[22]^ | F | 30 | NO | Yes | NO | NO | Yes | AChR | ADM: 20%; APB: 22%; FCR: 47%; TAM:  22% | Partial Remission | No |
| Scola R H et al.(2003)^[23]^ | F | 30 | NO | NO | NO | NO | Yes | AChR | Ulnar nerve: 12% with 3 Hz | Remission | No |
| de Carvalho M et al.(2006)^[24]^ | F | 38 | NO | NO | NO | NO | Yes | AChR | ADM: 18%(right), 13%(left); APB: 38%  (right) and 31% (left); right trapezius:  9%; right anconeus: 29% with 2 Hz | Remission | Yes |
| Renard D et al(2008)^[25]^ | F | 24 | Yes | Yes | NO | NO | Yes | AChR | Ulnar and median nerves:<10% (bilat  erally); radial nerve: 23% | Remission | No |
| Fearon C et al.(2015)^[26]^ | M | 47 | Yes | Yes | NO | NO | Yes | AChR | APB: 31% with 3 Hz | Remission | No |
| Jian Fan et al.(2017)^[27]^ | M | 42 | Yes | Yes | NO | NO | Yes | Titin/RyR | Deltoid muscle: 36%; ADM: 46%  with 3 Hz | Remission | No |
| Jian Fan et al.(2017) | M | 64 | Yes | Yes | NO | NO | Yes | Titin/RyR | Deltoid muscle: 6%; ADM: −2%  with 3 Hz | Remission | No |
| Jian Fan et al.(2017) | M | 62 | Yes | Yes | NO | NO | Yes | Titin | Deltoid muscle: 28%; APB: 9%; ADM:  55% with 3 Hz | Remission | Yes |
| Jian Fan et al.(2017) | F | 54 | Yes | NO | NO | NO | Yes | AChR | Deltoid muscle: 26%; APB: 42%; ADM:  4% with 3 Hz | Remission | Yes |
| Sousa DC et al.(2017)^[28]^ | M | 57 | Yes | Yes | NO | NO | Yes | AChR/MuSK/LRP4(-) | Right median: 27% (AM), 27% (AR); left median: 10% (AM), 16% (AR); spinal accessory: 18% (AM), 23% (AR); facial nerves: 16% (AM), 22% (AR) with 2 Hz | Remission | No |
| Bolz, S et al.(2018)^[29]^ | F | 42 | NO | NO | NO | Yes | Yes | AChR,Titin | abnormal decrement | Remission | Yes |
| Lauletta, A et al.(2023)^[30]^ | M | 47 | NO | NO | NO | NO | Yes | AChR,Titin | decremental response in both proximal and distal muscles. | Remission | Yes |
| Alanazy, M H et al.(2022)^[31]^ | F | 35 | Yes | NO | NO | Yes | Yes | AChR | decrement of 15% recorded from the trapezius muscle | Remission | Yes |
| Cao, M L et al.(2024)^[32]^ | F | 52 | NO | NO | NO | NO | Yes | AChR | Right ORB OCULI: 20.7%; right trape  zius: 24.4%; right ADM: 19.5%; right  brachioradialis: 23.2%; right APB 35.4%  with 3 Hz | Remission | No |

Supplementary Table 2 : Demographics and clinical characteristics of 39 patients

| **Characteristic** | **Thymoma association** | | ***p*-value** |
| --- | --- | --- | --- |
|  | **Non-TAMG**, n = 11^1^ | **TAMG**,  n = 28^1^ |  |
| **Age** | 50 ± 17 | 47 ± 12 | 0.609^2^ |
| **Sex** |  |  | >0.999^3^ |
| F | 6 (54.5%) | 16 (57.1%) |  |
| M | 5 (45.5%) | 12 (42.9%) |  |
| **ocular muscles** |  |  | 0.798^4^ |
| NO | 5 (45.5%) | 14 (50.0%) |  |
| Yes | 6 (54.5%) | 14 (50.0%) |  |
| **bulbar muscles** |  |  | **0.004**^3^ |
| NO | 4 (36.4%) | 24 (85.7%) |  |
| Yes | 7 (63.6%) | 4 (14.3%) |  |
| **neck muscles** |  |  | 0.609^3^ |
| NO | 9 (81.8%) | 25 (89.3%) |  |
| Yes | 2 (18.2%) | 3 (10.7%) |  |
| **respiratory muscles** |  |  | 0.296^3^ |
| NO | 11 (100.0%) | 23 (82.1%) |  |
| Yes | 0 (0.0%) | 5 (17.9%) |  |
| **Antibodies relative to MG** |  |  | 0.050^3^ |
| AchR | 7 (63.6%) | 17 (60.7%) |  |
| AchR/GAD | 0 (0.0%) | 1 (3.6%) |  |
| AchR/RYR/Titin | 0 (0.0%) | 1 (3.6%) |  |
| AchR/Titin | 0 (0.0%) | 6 (21.4%) |  |
| Agrin | 0 (0.0%) | 1 (3.6%) |  |
| ANA | 0 (0.0%) | 1 (3.6%) |  |
| negative | 2 (18.2%) | 0 (0.0%) |  |
| Titin | 0 (0.0%) | 1 (3.6%) |  |
| Titin/RyR | 2 (18.2%) | 0 (0.0%) |  |
| **Prognosis** |  |  | 0.726^3^ |
| Death | 0 (0.0%) | 3 (10.7%) |  |
| Partial Remission | 2 (18.2%) | 6 (21.4%) |  |
| Remission | 9 (81.8%) | 19 (67.9%) |  |
| ^1^Mean ± SD; n (%) | | | |
| ^2^Welch Two Sample t-test | | | |
| ^3^Fisher's exact test | | | |
| ^4^Pearson's Chi-squared test | | | |
| Th, thymoma. N, non-thymoma. | | | |

**Reference**

[1] Su M, Luo Q, Wu Z, et al. Thymoma-associated autoimmune encephalitis with myasthenia gravis: Case series and literature review[J]. CNS Neurosci Ther, 2024,30(2):e14568.

[2] Monstad S E, Nostbakken J K, Vedeler C A. CRMP5 antibodies found in a patient with limbic encephalitis and myasthenia gravis[J]. J Neurol Neurosurg Psychiatry, 2009,80(2):241-242.

[3] Evoli A, Lo M M, Marra R, et al. Multiple paraneoplastic diseases associated with thymoma[J]. Neuromuscul Disord, 1999,9(8):601-603.

[4] Jin Z S, Tao X R, Wang Z X. A case report of dermatomyositis mimicking myasthenia gravis[J]. Medicine (Baltimore), 2023,102(50):e36234.

[5] Lauletta A, Fionda L, Merlonghi G, et al. Distal upper limb involvement in myasthenia-myositis association[J]. Neurol Sci, 2023,44(2):719-722.

[6] Wang S, Yang H, Guo R, et al. Antibodies to Full-Length Agrin Protein in Chinese Patients With Myasthenia Gravis[J]. Front Immunol, 2021,12:753247.

[7] Furuta C, Yano M, Numanami H, et al. A case of thymoma-associated multiorgan autoimmunity including polymyositis and myocarditis[J]. Surg Case Rep, 2021,7(1):226.

[8] Muralidhar R Y, Parida S, Jaiswal S K, et al. Nocardiosis-an uncommon infection in patients with myasthenia gravis: report of three cases and review of literature[J]. BMJ Case Rep, 2020,13(12).

[9] Mehta M P, Sokol L L. The case of a 30-year-old man with subacute gait instability, weakness, and muscle spasms[J]. Ann Clin Transl Neurol, 2020,7(12):2535-2537.

[10] Bui H, Helms J L, Sierra-Hoffman M, et al. Thymoma Causing Bilateral Upper Extremity Deep Vein Thrombosis[J]. Respir Med Case Rep, 2020,30:101049.

[11] Dahal S, Bhandari N, Dhakal P, et al. A case of thymoma in myasthenia gravis: Successful outcome after thymectomy[J]. Int J Surg Case Rep, 2019,65:229-232.

[12] Fukushima K, Sato T, Mitsuhashi S, et al. Isaacs' syndrome associated with myasthenia gravis, showing remission after cytoreductive surgery of pleural recurrence of thymoma[J]. Neuromuscul Disord, 2006,16(11):763-765.

[13] Wakayama Y, Ohbu S, Machida H. Myasthenia gravis, muscle twitch, hyperhidrosis and limb pain associated with thymoma: proposal of possible new myasthenic syndrome[J]. Tohoku J Exp Med, 1991,164(4):285-291.

[14] Haiyan L, Qianqian Q, Jiejing S, et al. [Granulomatous Myositis Associated with Myasthenia Gravis: A Rare Case Report and Literature Review][J]. Journal of Rare and Uncommon Diseases, 2022,29(6).

[15] Yun J, Shifang H, Hua Z, et al. [The coexistence of myasthenia gravis, inflammatory myopathy in patients with thymoma—Three case reports and literature review][J]. Chin J Neuroimmunol & Neurol, 2024,31(01):1-7.

[16] Ueno T, Sato N, Kon T, et al. Progressive multifocal leukoencephalopathy associated with thymoma with immunodeficiency: a case report and literature review[J]. BMC Neurol, 2018,18(1):37.

[17] Uncini A, di Guglielmo G, di Muzio A, et al. Distal myasthenia gravis and sensory neuronopathy with anti-50 kDa antibody mimicking sensory-motor neuropathy[J]. J Neurol Neurosurg Psychiatry, 1997,63(3):414-415.

[18] Janssen J C, Larner A J, Harris J, et al. Myasthenic hand[J]. Neurology, 1998,51(3):913-914.

[19] Nations S P, Wolfe G I, Amato A A, et al. Distal myasthenia gravis[J]. Neurology, 1999,52(3):632-634.

[20] Gilad R, Sadeh M. Bilateral foot drop as a manifestation of myasthenia gravis[J]. J Clin Neuromuscul Dis, 2000,2(1):23.

[21] Musser W S, Barbano R L, Thornton C A, et al. Distal myasthenia gravis with a decrement, an increment, and denervation[J]. J Clin Neuromuscul Dis, 2001,3(1):16-19.

[22] Karacostas D, Mavromatis I, Georgakoudas G, et al. Isolated distal hand weakness as the only presenting symptom of myasthenia gravis[J]. Eur J Neurol, 2002,9(4):429-430.

[23] Scola R H, Iwamoto F M, Mainardi M A, et al. [Distal myasthenia gravis: case report][J]. Arq Neuropsiquiatr, 2003,61(1):119-120.

[24] de Carvalho M, Geraldes R. Longstanding right-hand weakness in a patient with myasthenia gravis[J]. Muscle Nerve, 2006,34(5):670-671.

[25] Renard D, Castelnovo G, Labauge P. Distal myasthenia gravis[J]. Acta Neurol Belg, 2008,108(3):107-108.

[26] Fearon C, Mullins G, Reid V, et al. Distal myasthenia gravis presenting as isolated distal myopathy[J]. Muscle Nerve, 2015,52(2):308-309.

[27] Jian F, Wang H B, Chen N, et al. [Observation of clinical and electrophysiological features in patients with distal myasthenia gravis][J]. Zhonghua Yi Xue Za Zhi, 2017,97(37):2894-2897.

[28] Sousa D C, Viana P, Leal I, et al. Ophthalmoparesis and unilateral finger flexor muscle weakness in seronegative myasthenia gravis[J]. Can J Ophthalmol, 2017,52(6):e213-e216.

[29] Bolz S, Totzeck A, Amann K, et al. CIDP, myasthenia gravis, and membranous glomerulonephritis - three autoimmune disorders in one patient: a case report[J]. BMC Neurol, 2018,18(1):113.

[30] Lauletta A, Fionda L, Merlonghi G, et al. Distal upper limb involvement in myasthenia-myositis association[J]. Neurol Sci, 2023,44(2):719-722.

[31] Alanazy M H, Alkhalidi H. Finger Flexor Weakness in Myasthenia Gravis[J]. J Coll Physicians Surg Pak, 2022,32(12):SS168-SS170.

[32] Cao M L, Qin Y X, Shao N, et al. Delayed diagnosis of distal myasthenia gravis: a case report[J]. J Med Case Rep, 2024,18(1):550.
